# Automated quantitative gait analysis in animal models of movement disorders

**DOI:** 10.1186/1471-2202-11-92

**Published:** 2010-08-09

**Authors:** Caroline Vandeputte, Jean-Marc Taymans, Cindy Casteels, Frea Coun, Yicheng Ni, Koen Van Laere, Veerle Baekelandt

**Affiliations:** 1 Division of Nuclear Medicine, K.U. Leuven, Leuven, Belgium; 2 Laboratory for Neurobiology and Gene Therapy, Molecular Medicine, K.U. Leuven, Belgium; 3 Department of Radiology, K.U. Leuven, Leuven, Belgium

## Abstract

**Background:**

Accurate and reproducible behavioral tests in animal models are of major importance in the development and evaluation of new therapies for central nervous system disease. In this study we investigated for the first time gait parameters of rat models for Parkinson's disease (PD), Huntington's disease (HD) and stroke using the Catwalk method, a novel automated gait analysis test. Static and dynamic gait parameters were measured in all animal models, and these data were compared to readouts of established behavioral tests, such as the cylinder test in the PD and stroke rats and the rotarod tests for the HD group.

**Results:**

Hemiparkinsonian rats were generated by unilateral injection of the neurotoxin 6-hydroxydopamine in the striatum or in the medial forebrain bundle. For Huntington's disease, a transgenic rat model expressing a truncated huntingtin fragment with multiple CAG repeats was used. Thirdly, a stroke model was generated by a photothrombotic induced infarct in the right sensorimotor cortex. We found that multiple gait parameters were significantly altered in all three disease models compared to their respective controls. Behavioural deficits could be efficiently measured using the cylinder test in the PD and stroke animals, and in the case of the PD model, the deficits in gait essentially confirmed results obtained by the cylinder test. However, in the HD model and the stroke model the Catwalk analysis proved more sensitive than the rotarod test and also added new and more detailed information on specific gait parameters.

**Conclusion:**

The automated quantitative gait analysis test may be a useful tool to study both motor impairment and recovery associated with various neurological motor disorders.

## Background

Multiple neurodegenerative and vascular central nervous system (CNS) diseases are characterized by motor deficits. For many of these diseases, no satisfactory neuroprotective or neuroregenerative therapies are available thus far. Therefore, the development of appropriate animal models for CNS motor disorders and the adequate evaluation of novel therapies in these animal models is an active field of preclinical research. In addition to the emerging non-invasive molecular and anatomical imaging techniques to visualize and quantify deficits and recovery in these disorders, behavioral testing is frequently the primary experimental readout to assess therapeutic effects. For this reason, sensitive, reproducible, time-efficient and easily applicable behavioral tests for existing or newly generated animal models are warranted. The present study has focused on animal models for three different motor disorders: the 6-hydroxydopamine (6-OHDA) rat models of Parkinson's disease (PD), a transgenic rat model of Huntington's disease (HD) and a photothrombotic cortical lesion model of stroke.

PD is the most common neurodegenerative movement disorder whose hallmark feature is an extensive and progressive degeneration of dopaminergic neurons in the pars compacta of the substantia nigra [1]. Among the most widely used and longest established animal models of PD are the dopaminergic neuron lesion models employing 6-hydroxydopamine (6-OHDA) [2,3]. Of these, the unilateral infusion of 6-hydoxydopamine (6-OHDA) in the basal ganglia at the level of the striatum or ascending dopaminergic fibers leads to unilateral dopaminergic cell death and to motor deficits in the side of the body contralateral to the lesion [4].

Next to toxin-induced HD animal models such as the administration of 2,3-pyridinedicarboxylic acid (quinolinic acid), transgenic mouse and rat models have been developed [5]. In the present study we use a transgenic rat model with a mutated Huntingtin gene containing 51 CAG repeats [6].

Thirdly, several animal models have been designed to mimic human stroke as closely as possible in order to test possible reparative effects and neuroprotective therapies [7]. Here, we use a model of focal ischemia by the systemic injection of a photoactive dye (Rose Bengal) in combination with visible light irradiation of the sensorimotor cortex. As with the 6-OHDA model, this lesion is usually applied to only one brain hemisphere leading to left-right motor imbalances in the lesioned animal.

The Catwalk method is an automated and computerized gait-analysis technique that allows objective quantification of multiple static and dynamic gait parameters. This method has previously been evaluated for use in animal models of pain [8,9], sciatic nerve injury [10,11] and arthritis [12,13], but not yet in animal models of CNS based movement disorders. Therefore, the aim of the study is to evaluate the suitability and sensitivity of the Catwalk method for behavioral characterization of different animal models of PD, HD and stroke.

## Results

### a. 6-OHDA model

**Figure 1:**
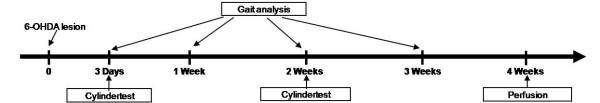
**Experiment time line for gait analysis in 6-OHDA PD model in rats**. Lesioned and control rat groups were prepared as described in materials and methods. Experimental groups were tested for motor deficits via automated gait analysis or via limb use asymmetry test (cylinder test) at indicated time points. Brains of animals were perfused at 4 weeks post-injection for histological analysis. *, measurement in striatal lesion groups only.

**Figure 2:**
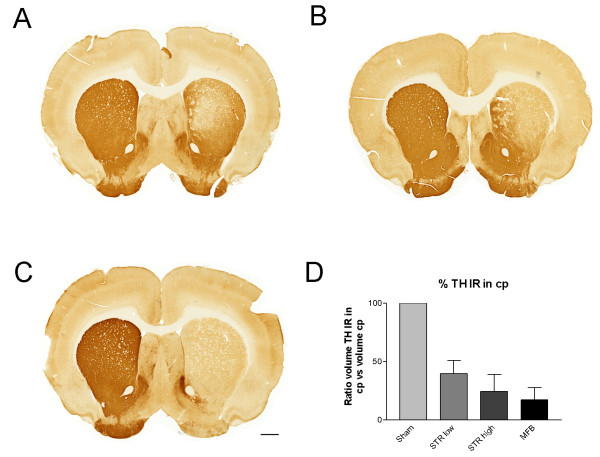
**Histological analysis of 6-OHDA lesioned rats**. Tyrosine hydroxylase (TH) immunohistochemistry reveals loss of TH staining in the striatum of the lesioned hemisphere following striatal lesion using 10 μg (A) or 20 μg (B) 6-0HDA or following 6-OHDA lesioning of the medial forebrain bundle (C). (D) Cavalieri quantification of the proportion of TH immunoreactivity retained in the caudate putamen for the separate 6-OHDA experimental groups, as well as the sham group. Data are shown as mean ± s.e.m. Scale bar, 1 mm.

A single dose of 6-OHDA (8 μg) was administered for the MFB group, two doses were tested for the STR injections (partial lesion group with 10 μg and full lesion group with 20 μg). Experimental groups were tested behaviorally in the cylinder test and using the catwalk method according to Fig. 1. Fig. 2A-C, show the histological confirmation of lesion size at 4 weeks post-injection after immunostaining of tyrosine hydroxylase (TH) positive dopaminergic fibers in the striatum. The loss of dopaminergic fibers in the caudate putamen was quantified stereologically via the Cavalieri method, and expressed as a percentage of total caudate putamen volume (Fig. 2D). The lesion size varied between 82.80% (s.e.m. 10.54) in the MFB group, 75.67% (s.e.m. 14.77) in the STR high group and 60.40% (s.e.m. 11.41) in the STR low group. Sham operated rats showed no loss in TH immunoreactivity (data not shown).

**Figure 3:**
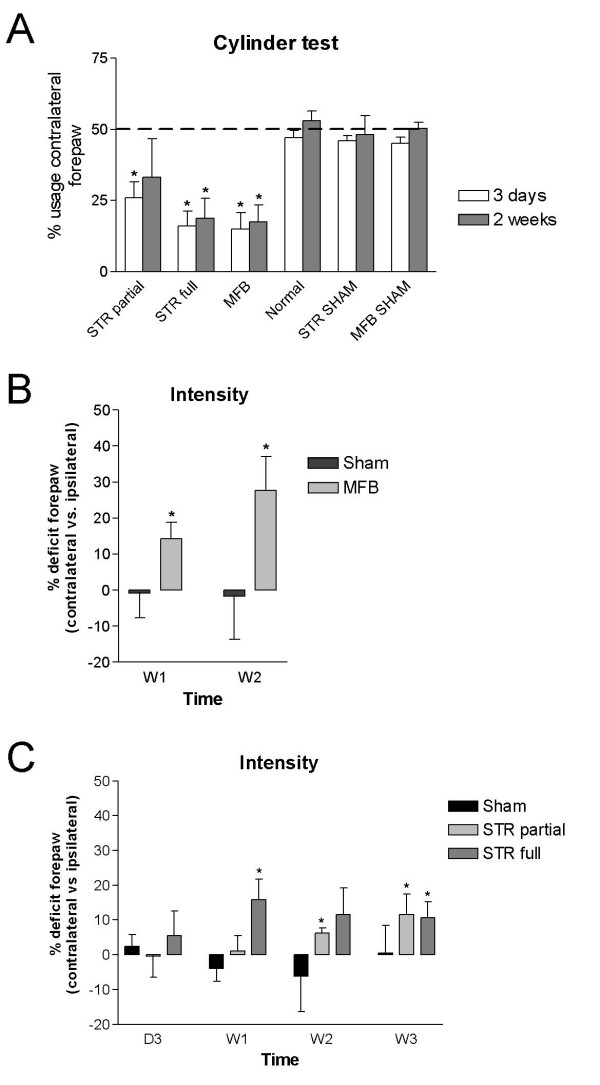
**Motor deficits following unilateral lesioning in rat basal ganglia using 6-OHDA**. (A) Proportion of limb use of 'lesioned' paws in 6-OHDA experimental groups as well as in normal rats and sham controls as measured in the limb-use asymmetry ('cylinder') test. Dashed line at 50% indicates the average expected paw usage in normal rats. (B) Deficits in pawprint intensity recorded via the catwalk method (expressed as % difference of contralateral vs. ipsilateral forepaws) in 6-OHDA medial forebrain bundle lesion or sham rats at 1 and 2 weeks post-lesion. (C) Deficits in pawprint intensity recorded via the catwalk method (expressed as % difference of contralateral vs. ipsilateral forepaws) in 6-OHDA striatal lesion or sham rats at 3 days, 1, 2 and 3 weeks post-lesion. Data are shown as mean ± s.e.m. * p < 0.05.

The cylinder test confirmed the lesioning behaviorally. Lesioned animals of the STR high and MFB groups showed a deficit in usage of the paw contralateral to the lesion both at 3 days and 2 weeks post-injection (Fig. 3A). This deficit was smaller in the STR low group and was statistically significant only for the early time point. Using the means and variances of the paw usage in the different groups, we determined that the minimal sample size required to detect significant differences (α < 0.05, 1-β > 0.8, effect size 25) was N = 5 for the STR low group and N < 3 for the 2 other 6-OHDA lesion groups.

Gait analysis parameters collected by the Catwalk system were first analyzed in the group showing the greatest extent of lesion, the MFB group. Significant contralateral ('lesioned' paw) vs. ipsilateral ('non-lesioned') differences were observed in the forepaws for the parameter 'intensity' at both time points (contralateral < ipsilateral, Fig. 3B). In the corresponding sham group, the 'intensity' parameter showed no significant contralateral-ipsilateral differences. Using the means and variances of the pawprint intensity imbalance measured for the MFB lesion group, we determined that the minimal sample size required to detect significant effects is N = 3. Other parameters showed no significant differences between paws contralateral to the lesion compared to paws ipsilateral to the lesion, with the exception of the parameter 'max area' (forepaws, at 1 week time point, contralateral < ipsilateral, ratio -12.1% s.e.m. 1.3, p < 0.05), and contact % (forepaws, at 2 week time point, contralatreral > ipsilateral, ratio + 43.5% s.e.m. 4.5, p < 0.05) (data not shown). No significant contralateral-ipsilateral differences were observed in the hindpaws in the MFB group.

In the experimental groups injected with 6-OHDA in the striatum, 'intensity' was significantly different between contralateral and ipsilateral forepaws starting at week 1 post-injection for the STR high 6-OHDA group and at 2 weeks for the STR low 6-OHDA group (Fig. 3C). Intensity (at 1 week, p < 0.01, and 2 weeks and 3 weeks, p < 0.05) as well as max area (at 2 weeks, p < 0.05) were also significantly different for hindpaws (contralateral < ipsilateral) in the STR high 6-OHDA group (data not shown). Sample size analysis using means and variances of pawprint intensity imbalance for these experimental groups yielded minimal sample sizes of N = 4-6. In the group having received a sham injection in the striatum, the 'intensity' parameter showed no significant contralateral-ipsilateral differences although variability was high (Fig. 3C, p = 0.18 - 0.56). No other parameters showed significant contralateral-ipsilateral differences (data not shown), and locomotor speed was not different between lesion and sham animals (Additional file 1).

### b. Rat transgenic model for Huntington's disease

Motor coordination and balance of 2- and 5 months old tgHD and WT rats were measured using the rotarod test. Throughout the study, there was no significant between-group difference in the latency to fall off the rotarod at all 5 speeds and ages tested (Additional file 2A), consistent with the asymptomatic description given in previous studies [14].

**Figure 4:**
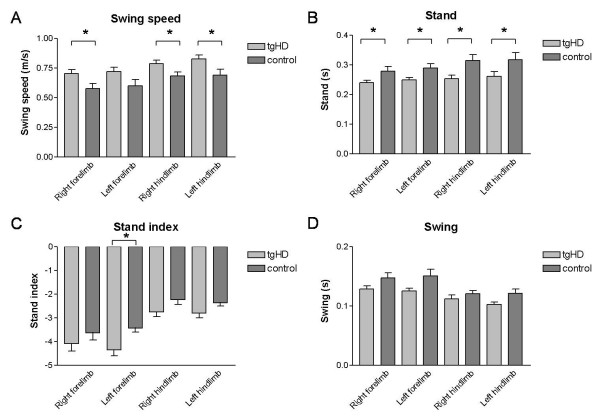
**Catwalk automated gait analysis test of tgHD and littermate control rats at two months of age**. (A) Shows the swing speed of all limbs for tgHD (light) and control rats (dark). (B) Duration of paw contact of all limbs for tgHD and control rats. (C) Speed at which the limbs lose contact with the glass plate for tgHD and control rats. (D) Swing time of all limbs for tgHD and control rats. Data are shown as mean ± s.e.m. * p < 0.03.

However, when gait abnormalities measured by the Catwalk method were assessed monthly, 2-month-old homozygous tgHD rats showed a significant increase in the swing speed of the hindpaws and right forepaw as compared to their wild-type littermates (Fig. 4A; all p < 0.03; min. sample size needed: N = 5-8), while the duration of paw contact was significantly decreased (Fig. 4B; all p < 0.02; min. sample size needed: N = 3-6). Locomotor speed was also significantly higher for 2 month old tgHD rats compared to controls (Additional file 1). Although other parameters did not reach statistical significance, this trend (p ≤ 0.10 for three out of four paws) was also seen in an increase in stand index (the speed at which the paws lose contact with the glass plate) (Fig. 4C) and a decrease in swing (the duration of no contact with the glass plate in a step cycle) (Fig. 4D). However, the differences in speed disappeared at the later time points investigated (Additional file 3).

**Figure 5:**
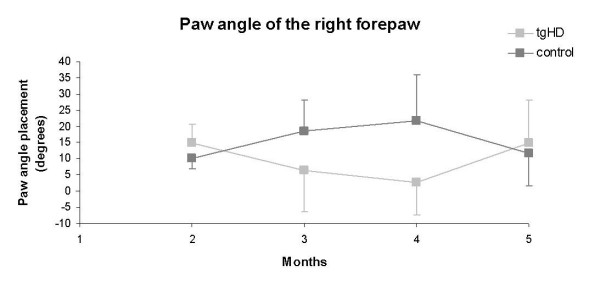
**Catwalk automated analysis of paw angle placement of tgHD and normal rats between 2 and 5 months of age**. At 3 and 4 months the right forepaw angle placement of the tgHD rats was deviated in comparison to the wild-types. Data are shown as mean ± s.e.m. * p < 0.03.

In addition, with increasing age, 3- and 4-month-old tgHD rats started to develop a deviating gait pattern, in which they placed the right forepaw under a smaller angle, i.e. more to the inside, than wild-type rats (Fig. 5; all p < 0.03; min. sample size needed: N = 2-7). This deviating gait pattern normalized at the age of 5 months. No significant differences in paw angle were detected between the different time points investigated within each group.

### c. Photothrombotic stroke model

**Figure 6:**
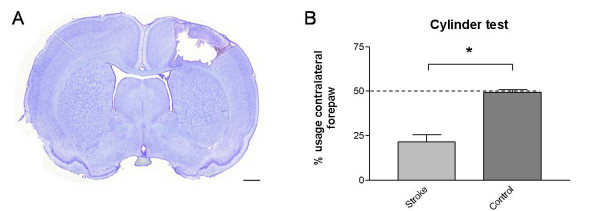
**Photothrombotic stroke model**. (A) Shows a representative cresylviolet staining of the photothrombotic stroke region. The limb use asymmetry test (B) showed a significant deficit in the usage of the forepaws contralateral to lesion site of stroke animals in comparison to the sham animals. Data are shown as mean ± s.e.m. * p = 0.029. Scale bar, 1 mm.

**Figure 7:**
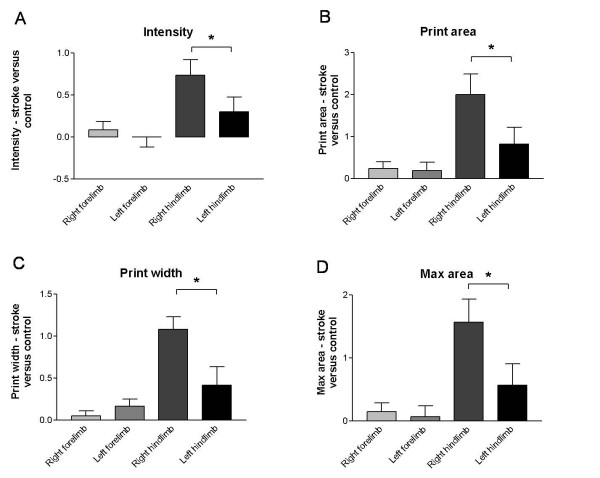
**Catwalk automated gait analysis of rats with a photothrombotic stroke compared to sham operated animals**. Experimental groups were prepared and submitted to catwalk gait analysis as described in Materials and Methods. Depicted are mean ratios (± s.e.m.) of pawprint intensity (A), print area (B), print width (C) and max area (D) parameters of stroke animals vs. sham operated animals for each limb. All parameters showed lower values for the contralateral hindlimbs in comparison to the ipsilateral hindlimbs. * p < 0.05.

As stroke model, we induced focal ischemia by the systemic injection of a photoactive dye (Rose Bengal) in combination with visible light irradiation of the sensorimotor cortex. A representative cresyl violet staining, revealing the stroke location of all photothrombotic rats is shown in Fig. 6A. All these animals were tested for their motor behavior and usage of the paws via the rotarod test, cylinder test and the Catwalk. The cylinder test showed a significant deficit in the usage of the forepaws contralateral to the lesion site 48 h after surgery (Fig. 6B; p = 0.029, minimal sample size determination: N < 3). Furthermore the results of the rotarod test suggested a discrepancy in usage, equilibrium and motor coordination of the stroke animals compared to the controls although no significance was reached (data not shown). 1 day after surgery the animals were tested on the Catwalk. Differences were found in intensity of the contralateral hindlimbs compared to the ipsilateral hindlimbs (Fig. 7A; p < 0.05). Linked to these results a significant difference was also observed in the print area, print width and max area parameters. All these parameters showed lower values for the contralateral hindlimbs in comparison to the ipsilateral hindlimbs (Fig. 7B, C and 7D; p < 0.05; min. sample size needed: N = 2-5). These differences were not present in the forepaws. No contralateral-ipsilateral asymmetry was observed in the sham operated animals, and locomotor speed was not different between lesion and sham animals (Additional file 1).

## Discussion

In this study we measured locomotor deficits in rat models of PD, HD and stroke using a novel automated gait analysis test, the catwalk method, and compared the results to deficits recorded using established behavioral motor tests. To the best of our knowledge, the present study is the first to evaluate these rat models of neurological disorders using an automated gait analysis concurrently measuring static and dynamic gait parameters.

The quantitative gait analysis used in the present study, the catwalk method, provides both static gait parameters as well as time-based information and a pressure parameter, in contrast to a conventional gait analysis test, such as the analysis of footprints obtained by allowing rodents to walk with inked paws along a sheet of paper, which provides information about static gait [15]. The 6-OHDA rat model for PD recapitulates one of the main features of PD, namely the loss of midbrain dopaminergic neurons. The loss of dopaminergic innervation of the caudate putamen leading to reduced activity of the basal ganglia thalamocortical motor circuit and motor deficits has been well characterized both in the rat 6-OHDA model as well as in the human disease [2,16,17]. One of the most sensitive rodent behavioral tests available to assess midbrain dopaminergic cell loss is the amphetamine induced rotation test; however a pharmacological challenge is necessary to induce the behavioral readout [18]. It has proved challenging in the field to develop behavioral readouts for dopaminergic cell loss without pharmacological challenge [2]. The 'cylinder' test (paw use asymmetry test) can be used to observe contralateral-ipsilateral motor imbalances in the forelimbs in unilaterally 6-OHDA lesioned animals without pharmacological challenge [19], as is confirmed in our experimental groups where the degree of impairment correlates to the degree of lesion (Fig. 2D). This contralateral-ipsilateral imbalance is also observed in the gait analysis, as the intensity of the lesioned forepaw is significantly lower compared to the non-lesioned paw, indicating that the 6-OHDA lesioned animals preferably use the non-lesioned paws to support their body weight. The amplitude of the intensity imbalance was consistent with the severity and type of lesion. Indeed, strongest measures were observed for the group showing the largest lesion, the MFB lesion group. In this group, strong paw intensity deficits were seen at 1 week post-lesion. On the contrary, striatal injections which yielded lower levels of dopaminergic fiber loss showed lower pawprint intensity deficits compared to the MFB group. A recent study has similarly analyzed 6-OHDA lesioned rats using the Catwalk gait analysis [20], including experimental groups with mild bilateral 6-OHDA lesions in the striatum combined with subthalamic nucleus stimulations. Because Vlamings et al. [20] performed bilateral lesions and our major observation is a contralateral-ipsilateral imbalance in pawprint intensity, it is difficult to draw parallels between both studies. We nevertheless observed that the contralateral-ipsilateral imbalances were bigger in the forepaws compared to the hindpaws, similar to differences of effects of forepaw vs hindpaw as observed by Vlamings et al. [20].

Interestingly, despite the availability of multiple parameters from the catwalk analysis, the only parameter which consistently yielded results corresponding to a unilateral motor deficit was the intensity parameter measured for the forepaws. This parameter is highly analogous to the paw usage parameter monitored in the cylinder test and we can therefore conclude that information obtained from motor behavior assessment of hemiparkinsonian rats using the catwalk test is comparable to that obtained by the cylinder test.

We have also monitored the onset and early progression of HD-like symptoms related to all motor systems affected in HD in a transgenic rat model of HD. In line with previous studies in this tgHD rat and in a knock-in mouse model of HD [14,21], gait analysis revealed a hyperkinetic profile early on in the pre-symptomatic stage. Whereas previous studies used the accelerod or open field monitoring [14,21], we have demonstrated by the aid of the Catwalk that the hyperkinetic profile in 2-month-old transgenic rats is more specifically reflected by increased swing speed and decreased paw contact of quasi all limbs, and lasts until the age of 3 months; the latter in accordance with a previous report [14]. 2-month-old transgenic rats also showed increased locomotor speed (Additional file 1), in line with previously described negative correlation between locomotor speed and stance duration [22]. This early hyperkinetic profile was not observed on the rotarod test used as a reference test in this study, allowing us to conclude that the catwalk method is more sensitive in measuring behavioural deficits in these animals than the rotarod test (Additional file 2A) [23]. Whether alternative behavioural tests, of which some perform better than the rotartod, e.g. the balance beam tasks, will also detect this hyperkinetic profile needs to be further studied ([24-26]).

Consistent with observations in humans, in which even years before the onset of overt motor symptoms such as chorea, subtle motor deficits are present (i.e alterations in finger-tapping rhythm and rate) [27,28], we also noticed in 3- and 4-month-old tgHD rats a deviant gait pattern, in which they placed the right forepaw more to the inside than WTs (Fig. 5). No significant differences in paw angle were observed between the time points of both groups.

Aberrations in paw placement have so far only been described in symptomatic mice transgenic for HD and in 3-NP-treated mice [15,29]. Specifically, R6/2 transgenic mice at 13-14 weeks of age exhibited a gait that lacked a normal, uniform, alternating right-left step pattern with inside paw placement of all limbs, although within 3-NP-treated mice, the paw placement angle of the hind limbs was more open [15,29]. The unilateral onset of this paw angle placement phenotype in the tgHD rats may be explained by reports suggesting that asymmetric striatal degeneration due to ventricle enlargement is not uncommon [6]. Whether the paw angle measured using the catwalk is indeed an early marker for motor deficits needs to be clarified in further longitudinal designs.

Thirdly, we used the Catwalk method to quantify disturbances in gait in rats with a unilateral photothrombotic lesion in the parietal sensorimotor cortex compared to sham operated animals. As for the tg HD animals, the rotarod test which we used as a reference test proved to be insensitive since only a fraction of the lesioned rats fell off the machine (Additional file 2B). As expected by the anatomofunctional location of the infarct, the rats showed a deficit in usage of the forepaw contralateral to the lesion 48 h after surgery as seen with the cylinder test. However, this test is not suitable for the detection of impairments in the hindpaws. In contrast, the gait analysis with the Catwalk method demonstrated a significant difference in the usage of the contralateral vs. ipsilateral hindlimbs. This was expressed by the 'intensity', 'print area', 'print width' and 'max area' parameters (Fig. 7). All these parameters showed that the lesioned animals put a higher pressure on the non-affected ipsilateral hindpaw than the contralateral hindpaw. This imbalance is likely due to a compensation of the animal to spare the affected paw and enforce the paw with non-injured motoric excitation. These results confirm data where cortical ablation lesions impaired the performances of the rats on a beam walking task caused by hindlimb deficits [30,31]. Our data therefore clearly demonstrate the advantage of using automated gait analysis for the study of cortically lesioned rats.

Although the present study has focused on gait analysis in rats, extension to mouse models is in principle possible. For example, robust effects on stride length during continuous locomotion following pyramidotomy of the cortical tract in adult mice have been observed [32,33]. Also, unsteady gait with strongly reduced paw print area for both fore- and hindpaws and reduced base of support for the hindpaws have been measured in a mouse model for Refsum disease [34]. Further research will be required to delineate gait deficits in mouse models of PD, HD or stroke.

## Conclusion

In the present study, we measured for the first time gait deficits in rat models of PD, HD and stroke using an automated quantitative gait analysis test and compared the results to motor deficits observed in reference tests. In the unilateral 6-OHDA lesion model for PD, the deficits in gait essentially confirmed results obtained by the cylinder test. However, in the HD model and the stroke model the Catwalk analysis proved more sensitive than the rotarod test and also added new and more detailed information on specific gait parameters. The automated quantitative gait analysis test may thus be a useful tool to study both motor impairment and recovery associated with various neurological motor disorders.

## Methods

### Animals and surgery

Animals were housed under 12 h light/12 h dark cycle with free access to food and water. All animal experiments were performed in accordance with national and international regulations and approved by the animal care and use committee of the K.U. Leuven. For the 6-OHDA and stroke model, animals were anesthetized before surgery with a ketamine (Ketalar^®^, Pfizer 60 mg/kg) and medetomidin (Domitor^®^, Orion Pharma, 0.4 mg/kg ip) cocktail. Anesthesia was reversed with atipamezol (Antisedan^®^, Orion Pharma, 1 mg/kg ip) at the end of the surgical procedures.

### 6-OHDA lesion

8 weeks old female Wistar rats (Janvier, Le Genest Saint Isle, France) were divided into different groups: animals with a unilateral lesion (striatal injection of 10 or 20 μg 6-OHDA (n = 5 per group), or medial forebrain bundle injection of 8 μg 6-OHDA (n = 5), all in 4 μl 0.9% NaCl containing 0.05% ascorbic acid, Sigma), sham-operated animals (n = 5 each for striatum and medial forebrain bundle, injected with 0.9% NaCl containing 0.05% ascorbic acid, Sigma Aldrich, Bornem) and control animals (n = 5). The striatal injections were performed at the following coordinates relative to Bregma: anteroposterior (AP) 0 mm, lateral (LAT) -2.8 mm and dorsoventral (DV) -5.5 mm [19]; coordinates for the medial forebrain bundle were AP -3.4 mm, LAT -1.6 mm and DV -8.2 mm [35]. Animals were placed in a stereotactic head frame (Stoelting, IL, USA) and the 6-OHDA or control solution was administered with a 30-gauge needle and a Hamilton syringe at a rate of 0.50 μl/min. The 6-OHDA solution was freshly prepared and protected from light to minimize oxidation.

### Rat transgenic model for Huntington's disease

We investigated 14 male homozygous transgenic (tgHD) rats (+/+) and 9 littermate controls (generous gift from Prof. Olaf Riess, University of Tübingen, Germany). The animals have a Sprague-Dawley background and all rats were genotyped by Southern blot analysis of genomic DNA extracted from the tail tips that were removed at the age of three weeks. At the age of approximately one month, the animals were transferred to the Molecular Small Animal Imaging center (MoSAIC) of the KULeuven, Belgium. Gait analysis was performed monthly for animals starting from the age of approximately 2 months until 5 months. In this stage, the animals are presymptomatic when evaluated with standard behavioral tools [14].

### Phototrombotic stroke model

8 week old male Wistar rats (n = 8, Janvier, Le Genest Saint Isle, France) were divided into two groups: 5 animals received a cortical stroke and 3 animals were sham-operated. Cortical ischemia was induced by the use of a photothrombotic stroke approach [36]. Photo illumination with green light (wavelength 540 nm; bandwidth 80 nm) was achieved using a Xenon lamp with heat-absorbing and green filters. The irradiation at intensity of 0.68W/cm^2 ^was directed with a 3 mm optic fiber, which was placed on the exposed skull above the right sensory-motorcortex. Photo illumination was performed during 20 min. after intravenous injection of the photosensitizer Rose Bengal (20 mg/kg) in a tail vein [37]. The photooxidation causes endothelial damage, platelet activation, and finally vascular occlusion [38]. We chose this model because of its possibility to induce an ischemic lesion in any desired cortical area and because it is one of the least invasive stroke models. The sham-operated animals underwent the same operation procedure however the photo illumination with the green light was omitted.

### Histological assessment and stereological quantification of the 6-OHDA lesion

6-OHDA rats were deeply anesthesized with pentobarbital and transcardially perfused with saline followed by ice cold 4% paraformaldehyde in phosphate buffered saline (PBS). After removal of the brain and overnight postfixation, 50 μm thick coronal sections were made using a vibratome (Microm, Walldorf, Germany). For immunohistochemistry floating sections were treated with 3% H_2_O_2 _to remove endogenous peroxidase activity, washed and incubated with an antibody raised against tyrosine hydroxylase (rabbit, 1:1000, Chemicon, Temecula, CA, USA) in 10% goat serum. After washing, the sections were incubated with biotinylated swine anti-rabbit antibody, followed by incubation with streptavidin horseradish peroxidase complex (DAKO). Immunoreactivity was visualized using 3,3' diaminobenzidine. In all rat brains, the needle tract was identified to verify correct positioning of the infusion needle.

The volume of lesion in the caudate putamen (TH-negative region) was determined by stereological investigation using the Cavalieri method (Stereoinvestigater; MicroBrightField, Magdeburg, Germany) as previously described [39]. The values for the lesioned hemisphere were expressed as a percentage of the total volume of the caudate putamen.

### Histological assessment of the stroke lesions

After behavioral testing, brains of stroke rats were perfused as described above for 6-OHDA rats. Cresyl violet staining was used to visualize the stroke region. For this, 50 μm thick coronal slices were mounted on microscope slides and covered with 0.5% cresyl violet for 5 min. Slices were rinsed with 0.05% acetic acid.

No histological analysis has been performed on the tgHD rats and wild-type littermates, as they were also used in other studies, where later time points were aimed.

### Behavioral tests

#### Limb use asymmetry test (Cylinder test)

The cylinder test was used as a reference test to quantify the use of the forelimbs of the 6-OHDA lesion and stroke animals in comparison to the sham-operated animals. The test was performed essentially as previously described [19]. Briefly, the animals were placed in a 20 cm wide transparent glass cylinder and a minimum of 25 contacts of the forepaw with the wall of the glass cylinder were recorded. The number of impaired forelimb contacts (the contacts of the limb on the side of the body contralateral to the lesioned or sham treated side of the brain) was expressed as a percentage of total forelimb contacts. This value was compared to the theoretical value of 50% whereby both paws are used equally. Control animals score around 50% in this test [40].

#### Rotarod

To assess effects on motor coordination between tgHD and wild-type animals, rats were trained to remain on a rotarod. All rats underwent a 3-day training program on a 7 cm diameter rotarod (Ugo Basile, Biological Research Apparatus, Varese, Italy). During the training period, each rat was placed on the rotarod at a constant speed (8 rotations per minute; rpm) for a maximum of 120 sec, and the latency to fall off the rotarod within this time period was recorded. Rats received four trials per day for three consecutive days, by which time a steady baseline level of performance was attained. The test consisted of two trials at 5 increasing speed levels, ranging from 8 rpm to 16 rpm. The mean latency to fall of the rotarod at each speed level was recorded. The same training procedure was used for the animals that endured a photothrombotic stroke. All animals were trained for 3 days, and were then tested at increasing speeds the day after surgery.

#### Catwalk quantitative gait analysis test

The Catwalk™ is a video-based analysis system to assess gait in voluntarily walking mice or rats (Noldus, Wageningen, The Netherlands). The Catwalk system objectively measures various aspects of footfalls in a dynamic manner. The principle of this method is based on an optical technique. The light of a fluorescent tube is completely internally reflected in the glass walkway floor. When the animal crosses the walkway the light leaves the glass and illuminates only the areas of contact. In this way the different paw contacts are visualized. Based on the position, pressure, and surface area of each footfall, multiple parameters are calculated: intensity of the paws (signal depends on the degree of contact between a paw and the glass plate and increases with increasing pressure), print length (length (horizontal direction) of the complete print), print width (width (vertical direction) of the complete paw print), print area (surface area of the complete print), stand (duration of contact of a paw with the glass plate in a step cycle), swing (duration of no contact with the glass plate in a step cycle), swing speed (speed of the paw during swing), stand index (measure for the speed at which the paw loses contact with the glass plate), stride length (the distance between successive placements of the same paw), angle (estimate of the angle (in degrees) of the paw axis relative to the horizontal plane). Locomotor speed was determined by dividing the covered distance on the runway (cm) by the time needed to cross it [22].

Because data acquisition is dependent upon the animals walking across the runway, one issue we had to take into account in the present study is the altered mobility or motivation to walk in animal models of movement disorders. Indeed, we noted that disease model animals showed reduced motivation to cross the test runway upon repeated testing. However, we found that a single 2 minute acclimatization session prior to lesioning procedures (for 6-OHDA and stroke) or early in life (HD model) was sufficient for successful crossings of the runway during the experiment itself. For data collection, three runs per animal and time point were performed by placing the animal in front of the start zone of the catwalk runway. Analysis was performed on a minimum of 4 normal step sequence patterns in each uninterrupted run.

### Statistics

For the cylinder test of the 6-OHDA and sham injected animals, percentages of paw usage in each group were compared to the theoretical value of 50% using a one-tailed Wilcoxon signed rank test. In the comparisons of the unilaterally 6-OHDA lesioned animals, ratios of values from contralateral paws vs. ipsilateral paws were taken for all catwalk parameters and represented as percentage difference between both paws. These ratios were tested for significant difference from 0 using the one-tailed Wilcoxon signed rank test. Catwalk parameters collected from stroke animals were normalized for each paw using values collected from sham operated animals. The values were expressed relative to the control mean [(stroke/control mean)-1] and were tested for significant difference between contralateral paws and ipsilateral paws using the one-tailed Wilcoxon signed rank test. Behavioral outcomes of tgHD animals were compared to wild-type littermates using the non-parametric Mann-Whitney U test. Using the means and variances of the parameters measured in the different experiments, we determined the minimal sample size required to detect significant differences (Sample Size = (Zα + Zβ)^2^. σ^2^/δ^2^, statistical significance level α < 0.05, power 1-β > 0.8, one-tailed test, σ = standard deviation, δ = effect size). Conventional statistics were carried out using STATISTICA v8.0 (Statsoft, Tulsa, OK, USA) or Graphpad Prism 3.0 (San Diego, CA, USA). Statistical significance was set at p < 0.05.

## Authors' contributions

CV, JMT, FC, CC performed behavioural and histological analyses. YN and KVL provided advice on the experimental setup. CV, JMT, CC, KVL and VB designed the study and wrote the manuscript. All authors have read and approved the final manuscript. The authors declare no conflict of interest.

## Supplementary Material

Additional file 1 **Locomotor velocities for each experimental group**. Velocities are expressed as centimeters traveled per second. Results of statistical comparisons between model animals and respective control animals are given in column 4. Click here for file

Additional file 2 **Shows the results of the Rotarod test performed on tgHD (A) and photothrombotic stroke rats (B) compared to control animals**. Experimental groups were prepared and submitted to rotarod analysis as described in Methods. Shown are latency to fall off the rotarod at different rotation speeds (8-16 rpm) using a session time of 120 seconds. Data are shown as mean ± s.e.m., In B none of the control animals fell during the imparted session time. Click here for file

Additional file 3 **Catwalk automated gait analysis test of tgHD and littermate control rats at three and four months of age**. (A and B) Show the duration of paw contact of all limbs for tgHD (light) and control rats (dark). (C and D) represent the swing speed of the same animals. Click here for file
