# Supplementary material for: Automated quantitative gait analysis in animal models of movement disorders
Source: BMC Neurosci. 2010 Aug 9;11:92. doi: 10.1186/1471-2202-11-92 (PMC2924851; doi:10.1186/1471-2202-11-92)
Supplement: Additional file 1 — Locomotor velocities for each experimental group. Velocities are expressed as centimeters traveled per second. Results of statistical comparisons between model animals and respective control animals are given in column 4. [file 1471-2202-11-92-S1.DOCX]

| **Experimental group** | **Time point** | **Velocity (cm/s) ± s.e.m.** | **Statistical significance**  **(model vs ctrl)** |
| --- | --- | --- | --- |
| tgHD | 2 months | 44.75 ± 3.08 | p = 0.05 |
| Control |  | 36.19 ± 2.70 |  |
| tgHD | 3 months | 39.17 ± 2.23 | N.S. |
| Control |  | 36.31 ± 2.57 |  |
| tgHD | 4 months | 43.57 ± 1.62 | N.S. |
| Control |  | 38.32 ± 3.91 |  |
| tgHD | 5 months | 37.12 ± 3.01 | N.S. |
| Control |  | 31.94 ± 5.26 |  |
| Stroke |  | 35.32 ± 4.44 | N.S. |
| Sham |  | 36.51 ± 0.96 |  |
| 6-OHDA str low | 3 days | 43.59 + 2.99 | N.S. |
| 6-OHDA str high |  | 45.36 + 6.62 |  |
| Sham str |  | 40.44 + 3.64 |  |
| 6-OHDA str low | 1 week | 46.73 + 1.93 | N.S. |
| 6-OHDA str high |  | 44.84 + 7.16 |  |
| Sham str |  | 38.61 + 3.69 |  |
| 6-OHDA str low | 2 weeks | 49.34 + 1.35 | N.S. |
| 6-OHDA str high |  | 52.55 + 6.66 |  |
| Sham str |  | 44.55 + 1.81 |  |
| 6-OHDA str low | 3 weeks | 48.93 + 3.17 | N.S. |
| 6-OHDA str high |  | 47.26 + 2.17 |  |
| Sham str |  | 42.66 + 3.01 |  |
| 6-OHDA MFB | 1 week | 32.38 + 2.58 | N.S. |
| Sham MFB |  | 39.22 + 5.65 |  |
| 6-OHDA MFB | 2 weeks | 42.18 + 3.70 | N.S. |
| Sham MFB |  | 45.82 + 5.57 |  |

N.S.: Not significantly different
