# Supplementary figures and images for: Automated quantitative gait analysis in animal models of movement disorders
Source: BMC Neurosci. 2010 Aug 9;11:92. doi: 10.1186/1471-2202-11-92 (PMC2924851; doi:10.1186/1471-2202-11-92)

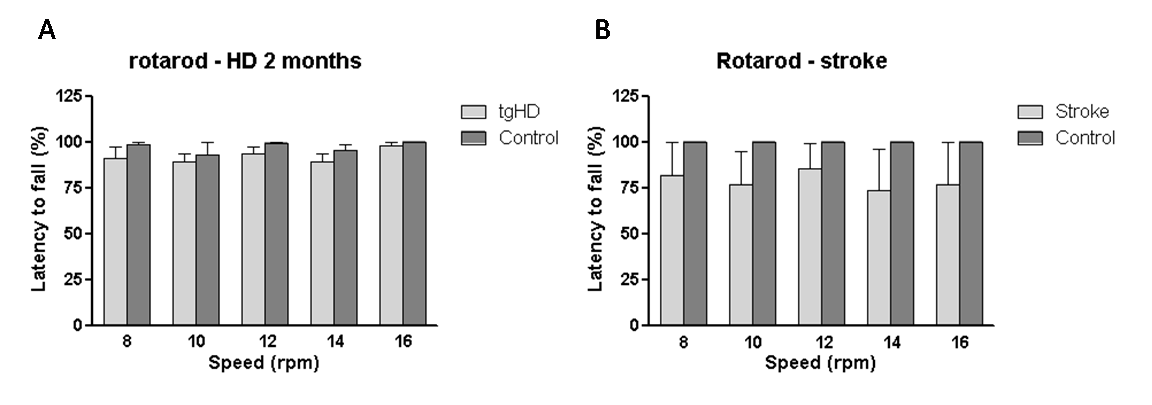

Supplement: Additional file 2 — Shows the results of the Rotarod test performed on tgHD (A) and photothrombotic stroke rats (B) compared to control animals. Experimental groups were prepared and submitted to rotarod analysis as described in Methods. Shown are latency to fall off the rotarod at different rotation speeds (8-16 rpm) using a session time of 120 seconds. Data are shown as mean ± s.e.m., In B none of the control animals fell during the imparted session time. [file 1471-2202-11-92-S2.TIFF]

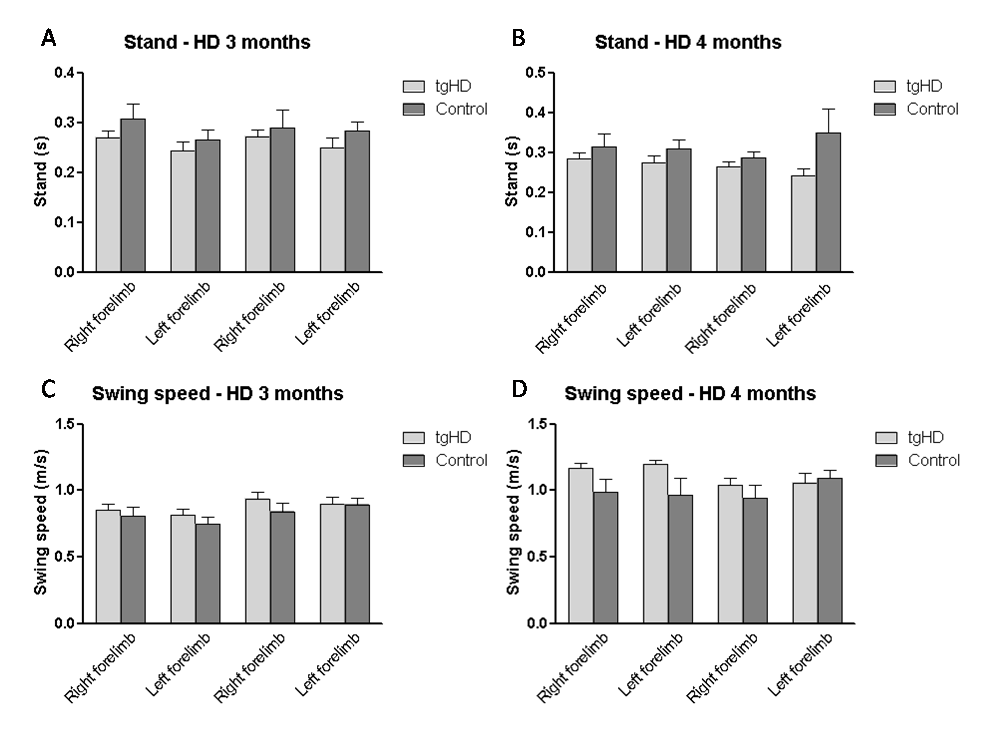

Supplement: Additional file 3 — Catwalk automated gait analysis test of tgHD and littermate control rats at three and four months of age. (A and B) Show the duration of paw contact of all limbs for tgHD (light) and control rats (dark). (C and D) represent the swing speed of the same animals. [file 1471-2202-11-92-S3.TIFF]
